# Laser-based Thickness Control in a Double-Side Polishing System for Silicon Wafers

**DOI:** 10.3390/s20061603

**Published:** 2020-03-13

**Authors:** Liang Zhu, Biao Mei, Weidong Zhu, Wei Li

**Affiliations:** 1 School of Mechanical Engineering, Zhejiang University, Hangzhou 310027, China; zhuliang@jsjd.cc (L.Z.); wdzhu@zju.edu.cn (W.Z.); liw@sfp.zju.edu.cn (W.L.); 2 Quanzhou Institute of Equipment manufacturing, Haixi Institutes, Chinese Academy of Sciences, Quanzhou 362216, China

**Keywords:** silicon wafer, double-side polishing system, laser probe, thickness control, B-spline fitting

## Abstract

Thickness control is a critical process of automated polishing of large and thin Si wafers in the semiconductor industry. In this paper, an elaborate double-side polishing (DSP) system is demonstrated, which has a polishing unit with feedback control of wafer thickness based on the scan data of a laser probe. Firstly, the mechanical structure, as well as the signal transmission and control of the DSP system, are discussed, in which the thickness feedback control is emphasized. Then, the precise positioning of the laser probe is explored to obtain the continuous and valid scan data of the wafer thickness. After that, a B-spline model is applied for the characterization of the wafer thickness function to provide the thickness control system with credible thickness deviation information. Finally, experiments of wafer-thickness evaluation and control are conducted on the presented DSP system. With the advisable number of control points in B-spline fitting, the thickness variation can be effectively controlled in wafer polishing with the DSP system, according to the experimental results of curve fitting and the statistical analysis of the experimental data.

## 1. Introduction

Silicon (Si) wafers are widely used for integrated circuits construction in the semiconductor industry. With the application of large-scale and ultra-large-scale integration devices, the Si wafers of larger sizes and thinner thickness are stringently needed, with which high output and well performance of integrated circuits can be expected. To achieve high cost-effectiveness in wafer processing, fine polishing or grinding systems, processes are studied by researchers. Eda et al. developed a single-step grinding system for Si wafers with 300mm diameter [1], which provides an integrated solution by using fixed abrasive to achieve the required surface roughness and global flatness. Sun et al. built a mathematical model of wafer shape for fine grinding of silicon wafers [2]. Lee et al. also presented a ϕ300mm wafer polishing system, and the effects of applied down-force and working temperature on surface characteristics were explored [3]. Schwandner et al. invented a method for the double-side polishing of a semiconductor wafer and presented the construction for carrying out the method [4]. Sun et al. established a predictive grinding-force model, as well as the relationship of subsurface crack depth and total normal grinding-force, to optimize the self-rotating grinding process for Si wafers [5]. Zhong et al. analyzed the effects of different pre-polishing processes on the site flatness values of the finished wafers in wafer polishing [6]. For semiconductor manufacturers, the improvement of quality, especially thickness uniformity of wafers, is an eternal pursuit, which demands precise evaluation and effective control of wafer thickness.

Thickness measurement and control are studied in many applications, such as mechanical devices and polymer material. Chowdhury measured oil-film thickness to establish a monitoring and control system for plain bearings [7]. Koberstein and Stein introduced the thickness measurement of the diffuse phase boundary by small-angle X-ray scattering for two-segmented polyurethane block copolymers [8]. Kita et al. discussed the applicability of using the brightness difference between the SiO_2_ and bare Si portions, on the scanning electron microscope (SEM) image of a nanometer-thin SiO_2_ layer on a Si wafer, to evaluate the relative thickness of the SiO_2_ layer [9]. Zarzycki et al. used an exponential two-layer light-material interaction model as an alternative to measuring the evaporate SiO_2_ thickness on Si wafers [10]. Yan et al. developed a scanning interferometry system by using the near-infrared low-coherence light to measure the surface profile, optical thickness and refractive index, of double-sided polished Si [11]. Lee and Joo proposed an optical interferometric method for the geometrical dimension measurement of the polished Si wafers [12]. Due to high accuracy, a laser scanner is also preferred for thickness measurements in some applications, such as medicine, archaeology and design. Tian et al. proposed an infrared-laser based thickness measurement method of Si substrates [13]. Pouli et al. investigated applying the depth profiling analysis of breakdown spectroscopy induced by nanosecond and femtosecond laser for the thickness measurement of thin organic protective coatings on historic metal objects [14]. Song et al. presented a solution to high-quality 3D reverse modeling on complex surfaces using a laser line-scanning sensor [15].

Moreover, various thickness control approaches were proposed. Kloeck et al. applied the electrochemical etch-stop for high-precision thickness control of Si membranes, which improves piezoresistive pressure sensors’ reproducibility [16]. Chung presented a similar method for controlling the thickness of single-crystal Si wafers in the aqueous tetramethylammonium hydroxide [17]. Zhu et al. explored the thickness uniformity control of a single layer to obtain uniform optical properties of a large-area soft X-ray multiplayer [18]. In this research, a laser probe is used as the tool for the thickness measurement of Si wafers.

Meanwhile, the laser probe is connected to a Programmable Logic Controller (PLC) via a personal computer (PC) to achieve the feedback control of wafer thickness. In wafer polishing, accurately characterizing the thickness and shape of Si wafers is the prerequisite of wafer thickness control. Thus, data fitting of the geometric profile based on the scan data from the laser probe is demanded.

Curve fitting is used to determine the parameters of a mathematical model that describes a set of usually noisy data in a way that minimizes the difference between fitting model and the data. Kinds of algorithms can be used for curve fitting. Florussen et al. applied ordinary polynomials functions for representing the geometric errors of multi-axis machines [19], in which appropriate polynomial order is obtained for every error component by analyzing the square root of the mean sum of squared errors. Kono et al. analyzed machine tool motion errors in the frequency domain to separate geometric errors from time-dependent errors [20]. However, due to leakage errors, the truncated Fourier series cannot accurately describe the component error in both of its ends because the measured error data rarely satisfy the periodic property. Ding et al. proposed an optimal modification approach to accurately modify tooth flank form errors [21], where higher-order polynomial functions of the cradle’s rotation angle are analytically treated as a motion element relative to a coordinate system. Kermarrec et al. considered the obtaining of Cartesian coordinates of control points by using a B-spline curve [22]. They also showed that a constant variance was accessed to all points of an object owing homogeneous properties, which does not affect the loss of efficiency of the least-squares solution.

In this paper, an elaborate double-side polishing system (DSP) is demonstrated for the fabrication of Si wafers. A novel feedback control scheme of the DSP, which integrates an optical coherence tomography (OCT) to shape the wafer thickness as the feedback, is stated. The precise location of the laser probe in the measurement of the wafer thickness is discussed. In addition, a B-spline representation is introduced for updating the wafer thickness model based on the measurement points on the wafer surface, which contributes to accurately estimate whether the polishing wafer reached the specified level of thickness. When the thickness function of the Si wafer is extracted from the corresponding scan data set, the residual errors can be treated as random errors. To verify the effectiveness of the elaborate DSP system and the novel thickness control method, experiments and result analysis are presented.

## 2. A Double-Side Polishing System for Si Wafers

In this section, the DSP system and laser-based thickness control (LTC) system for Si wafer processing are introduced. Communications among a laser probe, a PLC and a PC enables real-time detection, evaluation and location of the machining Si wafers, in which the wafer thickness is calculated by interference signal rebounded from the upper and lower surfaces of a wafer.

### 2.1. Polishing Unit with Feedback Control

**Figure 1:**
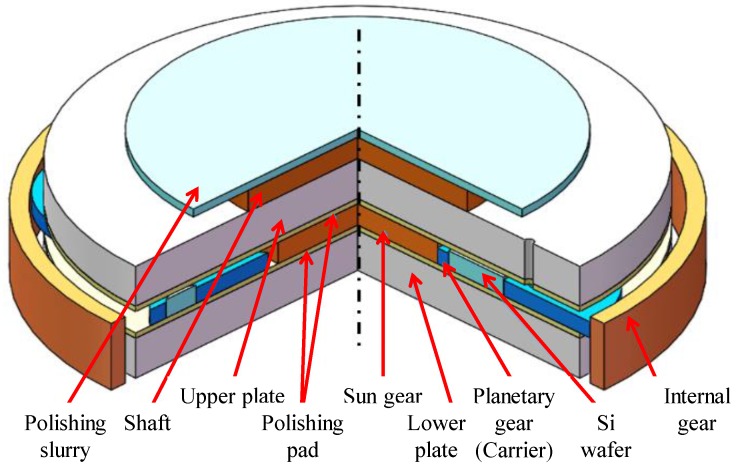
The mechanical structure of a double-side polishing machine for Si wafers.

An Si wafer, which has extensive application in printing integrated circuits and miniature integrated instruments, is one main component of the semiconductor apparatus. Polishing systems have been widely used in the fabrications of large-scale integrated circuits (LSI), very-large-scale integration (VLSI) and other semiconductors to obtain high-quality Si wafers. The mechanical structure of an elaborate wafer polishing machine is shown in Figure 1, which mainly consists of an upper plate, a lower plate, an outer internal gear, an inner sun gear and five planetary gears used as carriers for carrying 15 Si wafers. The internal gear is connected to the sun gear with the aid of the carriers.

**Figure 2:**
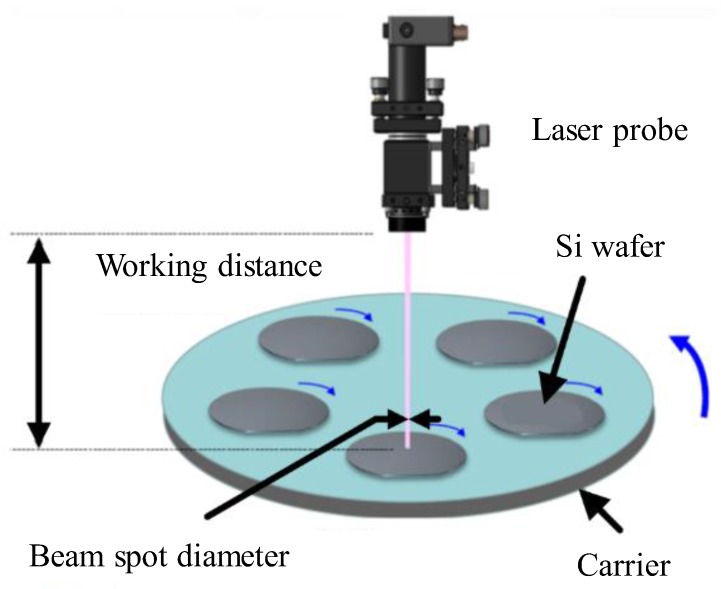
Wafer scanning with a laser probe.

The DSP system in Figure 1 aims at the Si wafers with 300mm diameter. In wafer processing, the sun gear and internal gear, as well as the upper and lower plates, rotate with preset speeds. Owing to the rotational speed difference between the two counter-rotating plates, the Si wafers on the carriers perform the planetary motion, which polishes the wafer surfaces to a specific thickness range. Meanwhile, the lens of Santec laser sensor is mounted on the upper plate and rotates along with it. A through-hole is manufactured on the top plate so that the laser beam can pass through. During polishing, wafers and the carrier rotate for laser scanning across the wafers, refer to Figure 2.

**Figure 3:**
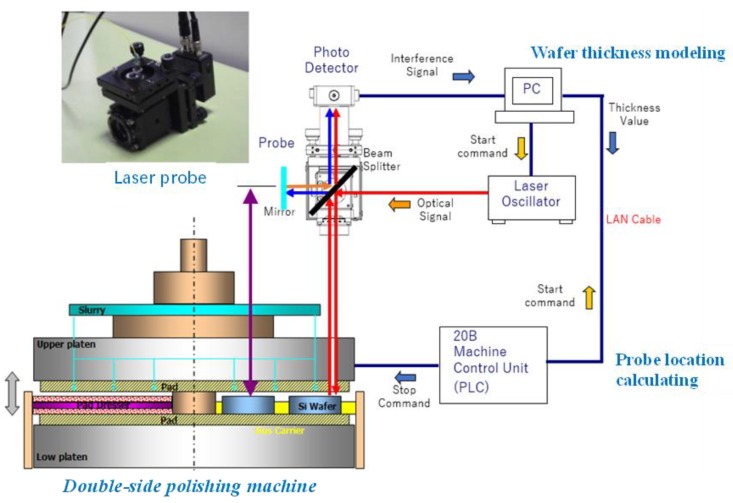
The laser-based thickness control (LTC) system of the double-side polishing (DSP) system.

The machining equipment also requires some software, the peripheral device, the control system, etc. The Q04UDEHCPU PLC from Mitsubishi is used for motion control of the mechanical structure of the wafer polishing machine. The MODBUS TCP is used as the communication medium between PC and PLC. An LTC system (see Figure 3) that consists of the thickness measurement, signal transmission and feedback control is constructed for polishing Si wafers with demanded technical indexes. In the LTC system, the thickness measurement of an Si wafer is achieved by using an OCT [23], which applies the basic principle of the weakly coherent light for detecting the time difference of the back reflection or scattering signals of the wafer’s upper and lower surfaces to calculate the wafer thickness. The main technical parameters of the thickness measurement system are shown in Table 1. By scanning of different positions at different times, the global shape of the wafer is obtained.

As shown in Figure 3, the laser probe moves over the moving wafer on the carrier to detect the thickness and shape with the laser beam. Then a photodetector delivers the received signals to the controlling PC for analysis. The detected scattered signal difference between double-side surfaces of the wafer is served as a valid signal to calculate the wafer thickness so that the global profile of the wafer is obtained. The real-time detection of the wafer profile is integrated into wafer processing. The PC would deliver the stop command to the PLC to stop the polishing processing once the profile of the machined wafer meets the preset thickness and surface shape.

### 2.2. Precise Location of Laser Probe 

The thickness measurement has two components: profile detection and probe location. The PC collects pulse information of the sun gear, internal gear and upper plate, as well as the elapsed time. Real-time physical coordinates of the carriers, wafers and laser probe are calculated based on the pulse information by comparing the timestamps of the PC and PLC. Along with the obtained thickness points, the real-time thickness-coordinate couples used as scan data can be obtained.

**Figure 4:**
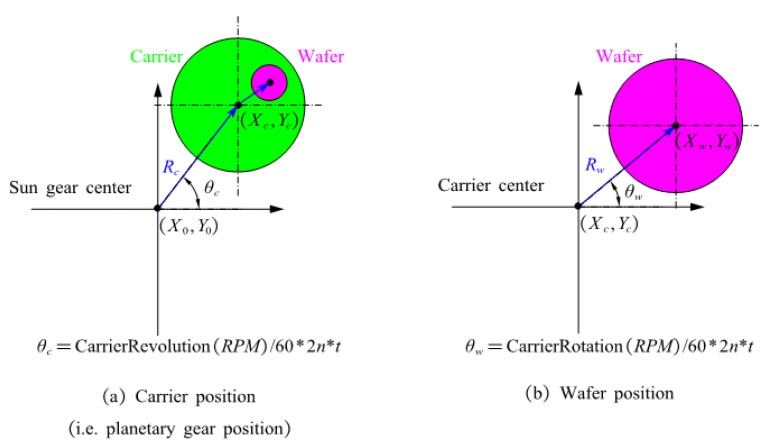
The calculation of carrier and wafer positions.

The calculation of the wafer position is illustrated in Figure 4. 

Firstly, the position coordinate of a carrier $\left(X_{c},Y_{c}\right)$ can be computed by

(1) $$\left\{ \begin{array}{l} X_{c}=X_{0}+R_{c}\times \cos(\theta_{c})\\ Y_{c}=Y_{0}+R_{c}\times \sin(\theta_{c}) \end{array}\right.$$

where $\left(X_{0},Y_{0}\right)$ is the coordinate of the sun gear, the default of which is (0,0); $R_{c}$ is the distance between the sun gear and planetary gear, which is a fixed parameter of the polishing equipment; $\theta_{c}$ is the real-time angle of the carrier relative to the sun gear, which can be calculated by the pulse information; the angle difference of adjacent carriers relative to the sun gear is 72°.

Similarly, the position coordinate of a wafer $\left(X_{w},Y_{w}\right)$ can be expressed by

(2) $$\left\{ \begin{array}{l} X_{w}=X_{c}+R_{w}\times \cos(\theta_{w})\\ Y_{w}=Y_{c}+R_{w}\times \sin(\theta_{w}) \end{array}\right.$$

where $R_{w}$ is the distance between the wafer and the carrier, which is also a fixed parameter of the polishing equipment; $\theta_{w}$ is the real-time angle of the wafer relative to the carrier, which can be calculated by the corresponding pulse information; The angle difference between adjacent wafers relative to the relevant carrier is 120°.

**Figure 5:**
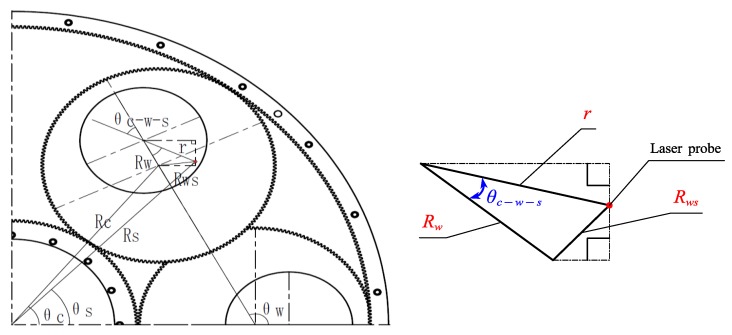
The calculation of the probe position.

Then, according to Figure 5, the position coordinate of the laser probe $\left(X_{s},Y_{s}\right)$ can be denoted by

(3) $$\left\{ \begin{array}{l} X_{s}=R_{s}\times \cos(\theta_{s})\\ Y_{s}=R_{s}\times \sin(\theta_{s}) \end{array}\right.$$

where $\theta_{s}$ is the real-time angle of the probe relative to the sun gear, which can be calculated by the obtained corresponding pulse information; $R_{s}$ is the distance between the laser probe and the center of the sun gear; $R_{s}$ is a fixed value since the laser probe is attached on the upper plate, and the installation position of the probe is determined by testing to obtain enough valid scan data.

Moreover, we can have the position coordinate of the laser probe relative to the wafer, assuming the wafer center is the origin.

(4) $$\left\{ \begin{array}{l} X_{ws}=r\times \cos(\theta_{c-w-s})\\ Y_{ws}=r\times \sin(\theta_{c-w-s}) \end{array}\right.$$

In Equation (4), r is the distance between the laser probe and the wafer, which is obtained by

(5) $$r=\sqrt{\Delta_{x}^{2}+\Delta_{y}^{2}}=\sqrt{{\left(X_{s}-X_{w}\right)}^{2}+{\left(Y_{s}-Y_{w}\right)}^{2}}$$

$\theta_{c-w-s}$ is the angle between the distances r and $R_{w}$ refers to the small triangle in Figure 5. With the law of cosines, the required angle can be easily calculated by

(6) $$\left\{ \begin{array}{l} \cos(\theta_{c-w-s})=\frac{R_{w}^{2}+r^{2}-R_{ws}^{2}}{2\times R_{w}\times r}\\ \theta_{c-w-s}=\cos^{-1}(\theta_{c-w-s}) \end{array}\right.$$

where $R_{ws}$ is the distance between the laser probe and the center of carrier, which can be calculated by

(7) $$R_{ws}=\sqrt{{\left(X_{c}-X_{w}\right)}^{2}+{\left(Y_{c}-Y_{w}\right)}^{2}}$$

The continuous data of the couples of thickness and (X,Y) coordinates are treated as the scan data. Only scan data passing through the wafer center is valid data, and other invalid data is removed. Meanwhile, we can further filter the out-of-tolerance scan data by PLC programming based on the preset thickness tolerance of the wafer. In addition, piecewise approximation can be applied for the pruning of the scan data.

## 3. Modeling and Evaluation of Wafer Thickness 

In wafer polishing with the DSP system, after the thickness measurement of the wafer, the measured data from the laser probe cannot directly be used for the thickness control. It is assumed that the exported unorganized data represents the profile of the wafer surface. Approximate curves should be fitted to the data of the wafer thickness to effectively obtain the representation. The most common method for curve fitting is the linear least-squares method, also called the polynomial least-squares. However, the B-spline model [24] is adopted to represent the thickness function of Si wafers considering the high-performance in geometric modeling.

### 3.1. B-spline Model for Characterizing Wafer Thickness Function 

In the field of computational geometry, B-spline is a useful model to represent freeform curves since a combination of B-splines can express any spline. The wafer thickness y(k) represented by a B-spline model is given below.

(8) $$y(k)=\left[\begin{array}{cccc} B_{0,d}(k) & B_{1,d}(k) & \cdots & B_{n,d}(k) \end{array}\right]\left[\begin{array}{c} C_{0}\\ C_{1}\\ \vdots \\ C_{n} \end{array}\right]$$

where $C_{i},(i=0,1,\cdots ,n)$ are control points, the number of which determines the flexibility of the thickness curve; $B_{i,d}(k)$ are basis functions of degree d of the B-spline concerning a knot vector $K=\left\{k_{0},k_{1},\cdots ,k_{n+d+1}\right\}$; the k is an estimated parameter to compute basis functions.

In this section, uniform B-spline basis functions, which are recursively denoted as below, are used.

(9) $$\left\{ \begin{array}{l} B_{i,0}(k)=\left\{ \begin{array}{ll} 1 & if\;k_{i}\leq k\leq k_{i+1}\\ 0 & otherwise \end{array}\right.\\ B_{i,d}(k)=\frac{k-k_{i}}{k_{i+d}-k_{i}}B_{i,d-1}\left(k\right)+\frac{k_{i+d+1}-k}{k_{i+d+1}-k_{i+1}}B_{i+1,d-1}\left(k\right) \end{array}\right.$$

In Equation (9), $k_{i}$ is the $i_{th}$ knot which is obtained by

(10) $$k_{i}=\{\begin{array}{cc} 0 & 0\leq i\leq d\\ \left(i-d\right)/\left(n+1-d\right) & d+1\leq i\leq n\\ 1 & n+1\leq i\leq n+d+1 \end{array}$$

To obtain a wafer thickness function based on the scan data, we fit the B-spline model to the scan data. The degree d is often chosen to be 3 to ensure C2 continuity. If we obtain a set of scan data $S=\left\{(x_{j},y_{j}),j=1,2,\cdots ,m\right\}$, the parameter $k_{j}$ can be computed by

(11) $$k_{j}=\frac{x_{j}-x_{init}}{x_{term}-x_{init}},j=0,1,\cdots ,m$$

where $x_{j}$ is of the $j_{th}$ scan point, $x_{term}$ and $x_{init}$ are of the terminative and initiatory scan data, respectively.

With Equations (9) and (11), we can obtain

(12) $$B=\left[\begin{array}{cccc} B_{0,d}(k_{0}) & B_{1,d}(k_{0}) & \cdots & B_{n,d}(k_{0})\\ B_{0,d}(k_{1}) & B_{1,d}(k_{1}) & \cdots & B_{n,d}(k_{1})\\ \vdots & \vdots & \ddots & \vdots \\ B_{0,d}(k_{m}) & B_{1,d}(k_{m}) & \cdots & B_{n,d}(k_{m}) \end{array}\right]$$

Thus, the thickness of a scan point, which is estimated by the fitted B-spline curve, can be denoted by

(13) $${\overset{⌢}{y}}_{j}=\left[\begin{array}{cccc} B_{0,d}(k_{j}) & B_{1,d}(k_{j}) & \cdots & B_{n,d}(k_{j}) \end{array}\right]\left[\begin{array}{c} C_{0}\\ C_{1}\\ \vdots \\ C_{n} \end{array}\right]$$

Intuitively, the residual errors of each point of the scan data set S can be obtained by

(14) $$e_{j}=y_{j}-{\overset{⌢}{y}}_{j},j=1,2,\cdots ,m$$

To evaluate the performance of characterizing wafer thickness function, the probability distribution parameters of the residual errors, i.e., mean value μ and standard deviation σ, are calculated by

(15) $$\mu =\frac{\sum_{j=0}^{m}e_{i}}{m+1},\sigma =\sqrt{\frac{\sum_{j=0}^{m}(e_{i}-\mu {)}^{2}}{m+1}}$$

### 3.2. Wafer Thickness Evaluation and Control Experiments 

In experiments, after we obtain the raw data of wafer thickness by using the laser probe of the DSP system shown in Figure 1, the B-spline representation is applied to build the wafer thickness model. Thus, the feedback control of the wafer thickness can be expected by using the LTC system in Figure 3. The procedure of the wafer thickness evaluation is briefly given as follows.

- Step 1: Obtain the raw thickness points of 0° and 90° measuring paths of Si wafer of “P-” type when scanning;
- Step 2: Explore the effects of the number of control points on the quality of the B-spline fitting of the raw thickness points, and choose an appropriate amount of control point;
- Step 3: Conduct the B-spline fitting of the raw thickness points based on deficient and superfluous control points to demonstrate the necessity of selecting the proper number of control points;
- Step 4: Contrast the results of the B-spline fitting and the biquadratic polynomial function to verify the effectiveness of the B-spline model on the characterization of the wafer thickness variation.

**Figure 6:**
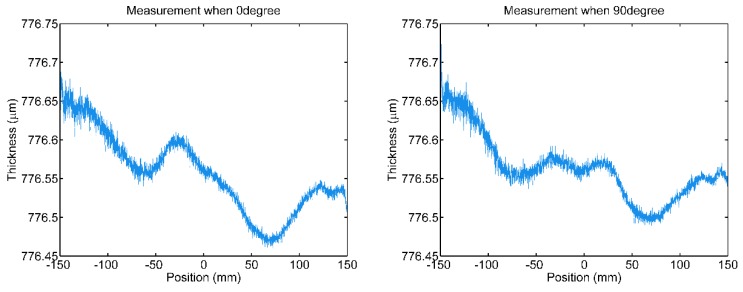
The scan data wrt. 0° and 90° measuring paths of Si wafer of “P-” type.

The scanning is conducted when polishing, and the raw thickness points in scan data concerning 0° and 90° measuring paths of a Si wafer of type “P-” are shown in Figure 6. The sampling frequency of the thickness measurement system is 3000 points per second. The preset thickness threshold is used to automatically remove the invalid data, including motion artifacts. In addition, the scan data which does not pass through the wafer center are also treated as invalid data and discarded. For fitting B-splines to the scan data of wafer thickness, the degree of the B-spline basis functions d, the number of the control points n+1 and the knot vector K should be determined in advance. As described above, the degree d is chosen to be 3, and a uniform knot vector is used. For the advisable flexibility of the B-spline model, the effects of the number of control points on the B-spline fitting are explored below.

**Figure 7:**
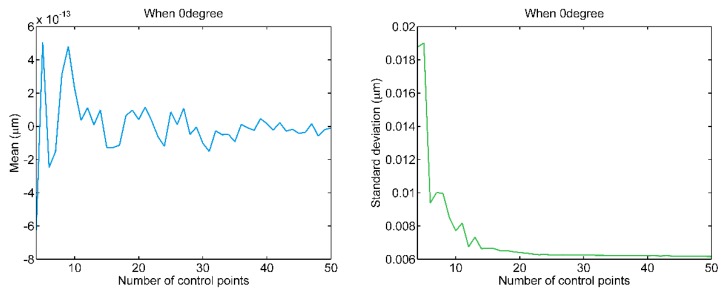

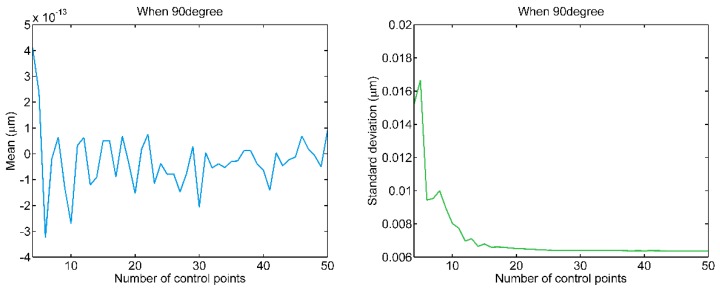
The means and standard deviations of residual errors wrt. number of control points.

The mean value and standard deviation of the residual errors from the B-spline fitting of the scanning date in Figure 6 are demonstrated in Figure 7. From the figures, it can be seen that the means are minimal regardless of the number of control points. At the same time, the standard deviations of the residual errors decrease with the increasing of the number of the control points from d+1 to 25 control points. After 25 control points, the increase in the number of control points induces very little change of the mean value and standard deviation. Therefore, the number of control points are chosen to be 25.

**Figure 8:**
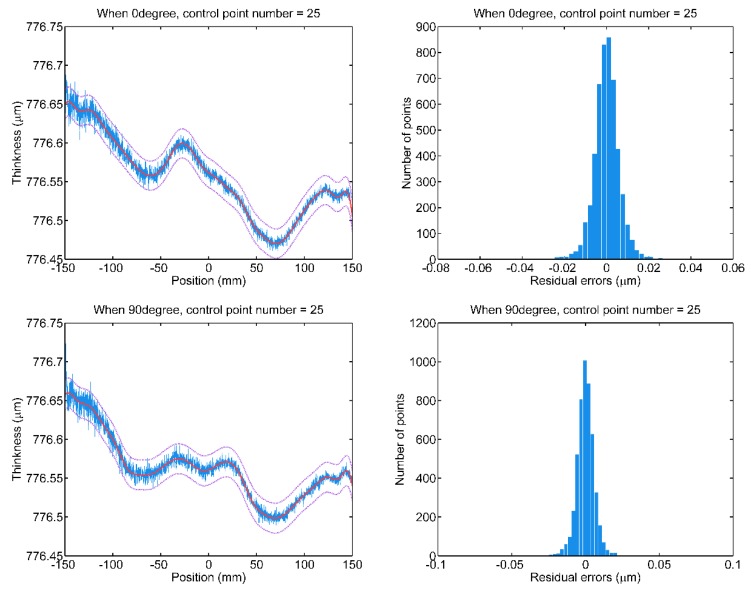
The B-spline fitting of scan data wrt. 0° and 90° measuring paths with a selected number of control points.

With the selected number of the control points, the B-spline fitting is conducted on two scan data sets of the Si wafer, to provide an accurate characterization of wafer thickness for thickness control. The fitting results of the 0° and 90° measuring paths are shown in Figure 8, respectively. The fitted curves that are drawn by a solid red line accurately characterize the thickness variations, and the residual errors approximate a zero-mean normal distribution. The two purple dotted curves denote the upper and lower ends of the thickness range, which is exactly ±3σ about the fitted curve. It means that 99.73% of the scanning points will be with the thickness range.

**Figure 9:**
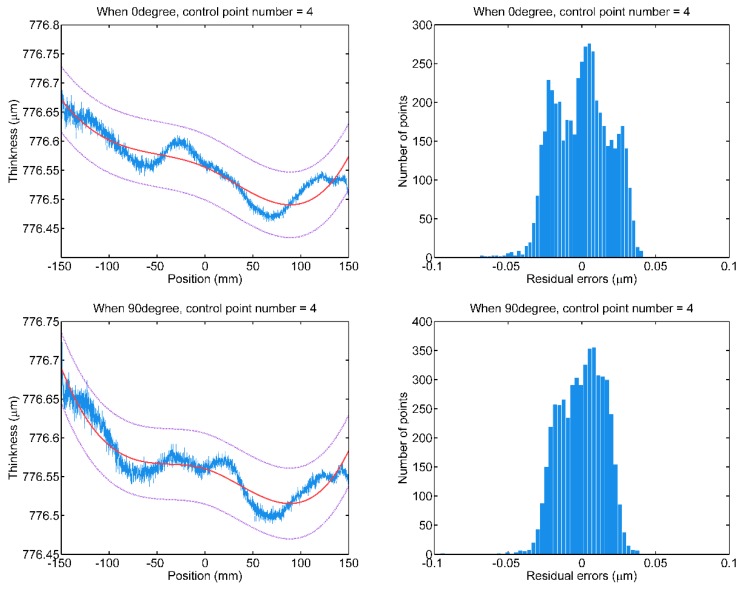
The B-spline fitting of scan data wrt. 0° and 90° measuring paths with deficient four control points.

To demonstrate the necessity of selecting the proper number of control points, the B-spline fittings based on deficient and superfluous control points are comparatively conducted on the scan data of a “P-” type wafer. Refer to Figure 9, when the B-spline model with deficient control points is fitted to the thickness scan data, the fitted curves cannot characterize the thickness variation, and some of the thickness is identified as random errors. Thus, the residual errors deviate from the normal distribution, and the standard deviations are large.

**Figure 10:**
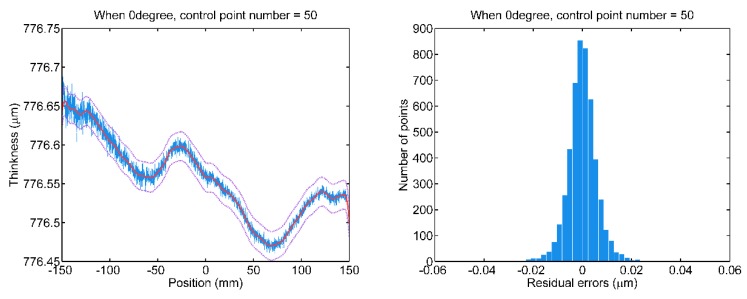

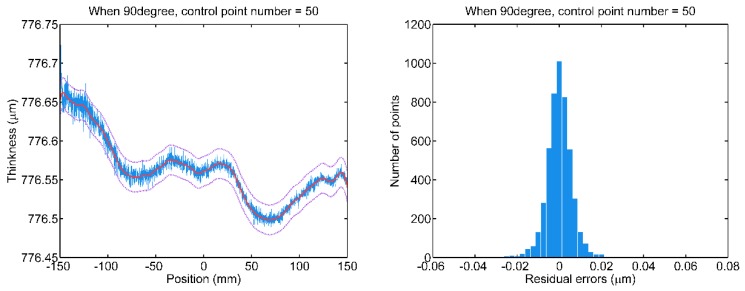
The B-spline fitting of scan data wrt. 0° and 90° measuring paths with superfluous 50 control points.

Refer to Figure 10, then the B-spline model with superfluous control points is fitted to the thickness scan data, in which some random errors are fitted by the B-spline model. Additionally, no obvious improvement concerning the residual error distribution.

**Figure 11:**
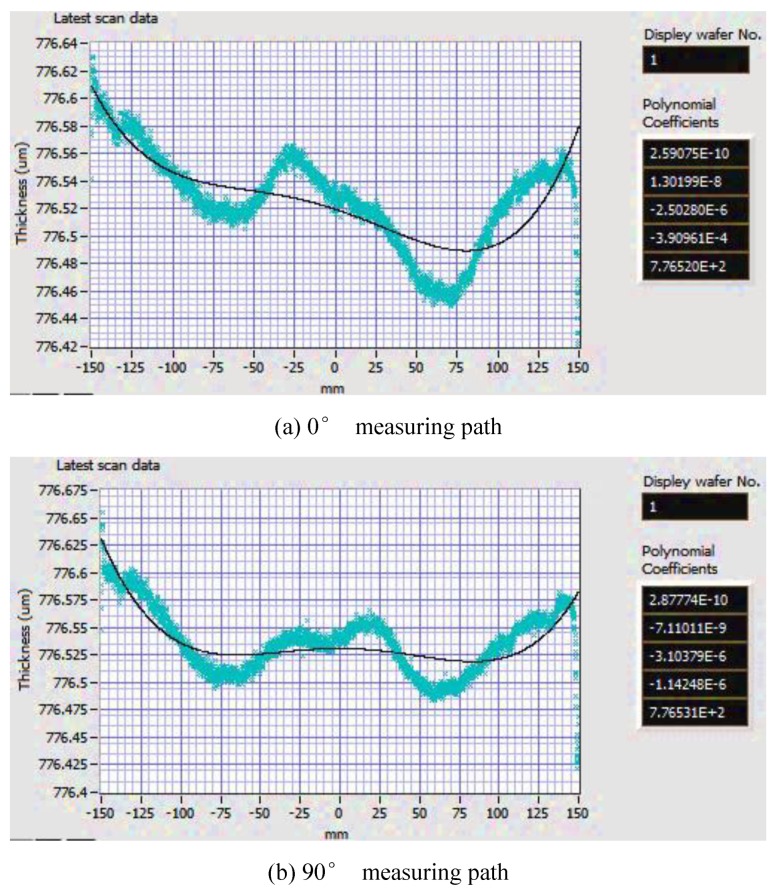
The biquadratic polynomial fitting of scan data wrt. 0° and 90° measuring paths.

Additionally, compared with the wafer thickness curves shown in Figure 11, which are fitted by the biquadratic polynomial function $Thinkness(X)=AX^{4}+BX^{3}+CX^{2}+DX+E$, the ones fitted by the B-spline model with a selected number of control points can accurately characterize the thickness variation. After the evaluation of the wafer thickness along the measuring paths, including 0° and 90° paths, the deviation of desired thickness from the fitted one provides the DSP system with the feedback for modifying the machining path of the DSP system. Then, the polishing unit with feedback control could be used to further polish the Si wafers. After thickness control, the root means square errors are intuitively and largely reduced. The magnitudes of the mean value and standard deviations could also be brought down, which can fulfill the quality requirement of Si wafers with 300mm in diameter.

## 4. Conclusions

For polishing large and thin Si wafers, an elaborate DSP system is demonstrated. The mechanical structure, especially the polishing unit, is discussed in detail. For effective wafer-thickness control, a laser probe is applied for thickness scanning, and a scheme of signal transmission and feedback control is constructed. To obtain continuous and valid scan data of the thickness, the mathematical model of the precise location of the laser probe is explored. Accurate thickness characterization of the wafer is the prerequisite of the thickness control. Thus, a B-spline model is used to represent the wafer thickness function, and the thickness deviation can be calculated for online machining compensation of the DSP system. In the B-spline fitting, the number of control points is adequately selected for excellent fitting performance. Experimental results show that the wafer thickness in Si wafer processing can be effectively controlled by using the presented DSP system in which the wafer thickness is evaluated by a B-spline model.

## Figures and Tables

**Table 1:** The main parameters concerning the thickness measurement system.

Parameter	Unit	Specification
Thickness measurement range	μm	3–1325
Working distance	mm	3–1000
Repeatability(1σ)	nm	≤35
Measurement frequency	Hz	30,000
Beam spot size	mm	Φ2.7
Operation temperature range	degC	25+/−5
Storage temperature range	degC	10–40
Storage humidity range	%	≤80
